# Is the Indicated Remedy an All-Sufficient?

**Published:** 1892-07

**Authors:** W. R. Bentley

**Affiliations:** Morristown, Indiana


### IS THE INDICATED REMEDY AN ALL-SUFFICENT?

W. R. Bentley, M. D., Morristown, Indiana.

(Read before Indiana Institute May, 1892.)

Wishing to throw in my mite along with others in favor of Homœopathy and the interest of our present session, I have concluded to report a few cases in confirmation of above.

Case I.—On July 13th, 1888, there came to my office a small, pale, emaciated, lame boy using crutches, as left foot could not be used owing to intense suffering.

He gave his name as Charles S——, aged twelve. The trouble appeared to be in the left hip, accompanied with intense pain in knee just beneath the patella.

*History.*—Mother died young with consumption; father living. When small, whilst playing with his father, riding on his back whilst his father would crawl on all fours, he fell off, hurting his left hip. Not much attention was given to it at the time, but as he grew older he was lame in this joint. For last two years has pain in left hip when receiving a jar.

Last year it began to pain him when walking or running. He says the pain was in the knee, just beneath the patella.

Last of May (1888) commenced to grow worse in left hip; it would give out or break down; pain dead in character, worse on motion, better from rest. Would have to put his hand on his knee to support himself or catch hold of something for support.

*Present Condition.*—Objective, pale; tired look; emaciated. Physical examination of limb revealed it smaller, apparently shortening; the limb was adducted; the toes of left foot resting on right; marked prominence of left hip as if dislocated; pressure around joint caused a dead aching referred to knee as well as to hip. Lordosis marked, disappearing on raising foot.

Touch caused spasmodic twitching of limb; pressure on inner side of knee between patella and joint caused dead aching in knee as well as hip. Subjective; pain heavy, dead aching in hip and also knee; compared to electricity playing between knee and hip; did not feel between knee and hip, but in knee and hip; worse on motion even of toes; worse from midnight until ten o'clock A. M.; then felt easier until three or four P. M.; worse windy, cold or rainy weather.

Would sleep until one A. M., then wake up, hip paining and feeling cold, also leg and foot. This coldness would last until he would get up and warm the limb by the fire; even in warm nights must get up and go to the fire and warm these parts.

He says limb jerks and feels like a knife were cutting along posterior part of thigh.

Leaders (flexors) won't go down; feel like hard cords. When felt they were jerking; adductors drawing the leg inward. Moving the limb feels like cutting the leg nearly off about hip. When he wakes up now the knee feels so stiff can hardly bend it. Has to bend it by degrees. All worse in after part of night.

In the morning aching deep in hip joint and knee, more especially knee. On trying to move it has to bend it by degrees, then jerk it right back to get relief. This has to be repeated frequently before he can bend the knee.

When he sits down it gets stiff and he has to move leg though it increases the pain.

Appetite, fair; bowels, fair condition; kidneys, about normal; sleeps good until midnight or one A. M., then suffers with pain and coldness.

Let the pathologist name the disease, but, as a Hahnemannian, I gave Rhus-tox.$^{1M}$ with decided relief, after first night, until August 8th, when my record shows this:

Pains more than for some time; pains at times severe; sharp aching pains; like needles sticking in hip and knee; sometimes in the leg. Most pain in knee right under patella, inner side. These pains come on just after sitting down, after moving. Last about five minutes; leave gradually.

Knee feels cold; wants it rubbed; not too hard. Does not dare to bear hard on knee cap.

Knee not so stiff as it was; can bend all at once, whilst formerly had to bend by degrees; twitching gone; jerking gone unless he hurts it. Last two mornings some desire to go to the fire to warm himself; cold over body; sleeping better; soreness in knee and hip in morning; bowels normal; appetite better.

*Physical Examination.*—Left limb seems to be two inches shorter than right, but measurement reveals no difference in length from anterior superior spinous process to internal malleolus. Shortening due to adduction and tilting up of hip and illeum. Lordosis marked, when he places the left foot down on floor flat has to bend right knee. Hip tender to pressure just above great trochanter, veins not so prominent as on first examination. Knee tender to touch at inner side of patella. Locates a spot here which is very tender and sore. Rhus-tox.$^{1M}$, as it had been worse just before a few days of wet weather.

Improvement continued uninterruptedly until in October, when he contracted the measles, but after getting over them still convalesced, until he dispensed with his crutches in January, using a cane to assist him in walking. By this time the adduction had given way to normal, except foot slightly turned out and a slight degree of shortening. Some pain yet in hip-joint, and a pointing as if it would break and run; knee seemed all right; limb increasing in size; boy feeling better every way. Owing to tendency to suppurate, I gave three powders of Hepar$^{3x}$, being highest I had at that time. This relieved tendency to suppuration, and it did not open for about eight months, when it broke and discharged pus in small quantity for about one month, then healed and remains to this day. The limb is shortened about one and one-half or two inches.

This patient had been treated by an allopath, using Morphine and other anodynes, both internally and externally, yet Rhus$^{1M}$ stopped all the pain, gave sleep, removed condition causing sickness, put the boy on road to recovery, and Hepar finished the work. To-day he is a trusted agent on C. H. & D. R. R.

CASE II.—Mr. Z. P., aged fifty-five, farmer, married, medium stature, black hair and eyes, fair skin.

December 22d, 1890, the above-named individual visited me, wanting treatment for a large sore on the dorsal surface of right hand.

*History.*—Four years ago there appeared on right dorsal surface, between second and third metacarpal, about one-half way between carpus and phalanges, a small, dry, whitish, scaly place. Dry scale came off, leaving a denuded surface, which has grown until at present about as large as a nickel five-cent piece. Circular in form.

*Objective.*—Edges or circumference elevated, whitish in color, hard to feel or touch; seemed to be of a hard, scaly nature. Centre has a papillary or granulated appearance, similar to a wart; darkish in color; movable by placing finger on top and shaking it.

Between this centre and outside raised zone a space, denuded, eaten below normal cutaneous surface; red and fiery, showing granular appearance from which exudes a pus-like discharge.

The external zone or raised ring was surrounded by a red inflamed zone about one inch wide. No particular pain on pressing on any part.

*Subjective.*—At times, especially evenings, a sharp, lancinating twinge frequently felt; worse in cold weather; lymphatics not inflamed. Has a rheumatic pain in right shoulder; worse at night; at times cannot lie on that side. Frequent urination for some years; has to urinate about twice during night; no pain, but sudden desire and trouble to retain longer; not sure of quantity; dull pain across loins and small of back. This combination, together with appearance of ulcer, gave me trouble in selection of remedy, so gave Sac.-lac., and promised to send medicine by mail. Hull's Jahr, under Kali-bich., gave a good description of ulcer and Hering's symptoms of same remedy covered remainder. I gave Kali-bich.$^{2cc}$, three powders, and Sac.-lac.

January 6th, 1891.—Reports feeling better; rheumatic pains in shoulder gone. Ulcer looking better; urinary symptoms better. Greater part of time has only to urinate once during night.

The hard raised ring surrounding ulcer at first examination is gone from two-thirds of circumference whilst remaining less raised and hard. Red zone, smaller and less inflamed. Centre covered with a dark, brownish scab, except right in centre, which has small, hard horny appearance, but not so large as when first seen. Looks better every way. Measurement three-quarter inch longest, five-eighth shortest diameters. Less of lancinating pains. Gave Sac-lac.

January 27th.—External raised ring gone; still dark brownish scab, but smaller; measurement five-eighth inch longest, half inch shortest diameters. Looks and feels better. Lancinating pains gone. Urinary symptoms better. Ulcer maturating. Sac-lac.

February 12th.—Ulcer covered with a brownish scab; measurements about same; urinary symptoms better; can retain urine without any trouble. As a stand-still in ulcer seemed to exist and no new or contrary indications, gave four powders Kali-bich.$^{2c}$ and Sac-lac.

Under this, steady improvement until in June, when ulcer completely healed. Saw patient a few days since, when all that could be seen was a slight discoloration remaining.

Considering that two or three allopathic physicians had failed and various other means were not efficient, we are rejoiced to see the silent force work so great a revolution.

CASE III.—Mrs. J. B., aged eighty-four, widow. April 30th, 1892, little granddaughter came to office stating grandma would like me to come over to see her. On entering the room she stated she had been coughing up blood. Asking her if she had preserved it she directed me to look on ground just outside of veranda. To my surprise I beheld a large amount of bright red blood partially clotted.

Returning to room and trying to get symptoms, I found first had been coughed before daybreak, repeated at intervals of ten to fifteen minutes. She had been well for some time. I could get no symptoms on which to base a prescription except blood bright red and mixed with mucus, raised by a very slight cough. On this I gave Ipec.$^{1m}$, three doses one hour apart; this controlled it for several hours when it began again worse than before. Sending for me again I found blood coughed up, darker, clotted, mind perfectly calm. The hemorrhage had become alarming by this time. Returning to office, consulted *Repertories* and *Materia Medica*. I selected Hamamelis, which I have in the 3x the highest I had, which after third dose controlled it nicely, leaving weakness as the only bad effect, from which care and good nursing has completely restored her.

Do these cases answer the question? To my mind, yes, connected with other experience. Each of you, my brother physicians, has had the same experience. Why should we resort to empiricism? Echo answers why?

*Discussion.*—Dr. L. W. Jordan, of Indianapolis, desired to praise the complete descriptions given.

Dr. W. B. Clarke, of Indianapolis, thought that the hip-joint case had not been a great success. A life may have been saved by nature, but the patient was practically a cripple. He called attention to the practice of Surgeon S. B. Parsons, St. Louis, who in the Children's Hospital there sees these cases, and aspirates the joint early. These cases are usually tuberculous, and require some mechanical and local treatment.

Dr. T. M Stewart, of Cincinnati, was emphatic in the belief that the case should have had a long splint and extension. Dr. Bentley closed by saying that the parents would allow nothing mechanical done, desiring only to protect the child against pain.

